# Health-Related Behaviours of Foreign Students Studying in Poland and Their Determinants: A Mixed-Methods Study

**DOI:** 10.3390/nu16081149

**Published:** 2024-04-12

**Authors:** Agnieszka Chrzan-Rodak, Jadwiga Bąk, Justyna Chałdaś-Majdańska, Michał Machul, Anna Obuchowska, Aleksandra Grzegorczyk, Magdalena Dziurka, Patrycja Ozdoba, Beata Dobrowolska

**Affiliations:** 1 Department of Family and Geriatric Nursing, Faculty of Health Sciences, Medical University of Lublin, 20-081 Lublin, Poland; agnieszka.chrzan-rodak@umlub.pl; 2 Upper Silesian Centre for Child and Mother’s Health, Medyków Street 16, 40-752 Katowice, Poland; jadwigabak25@gmail.com; 3 Faculty of Human Sciences, WSEI Academy, Projektowa 4 Street, 20-209 Lublin, Poland; justynachaldas@wp.pl; 4 Department of Holistic Care and Nursing Management, Faculty of Health Sciences, Medical University of Lublin, 20-081 Lublin, Poland; michal.machul@umlub.pl (M.M.); annaobuchowska2@gmail.com (A.O.); aleksandragrze.97@gmail.com (A.G.); magdalena.dziurka@umlub.pl (M.D.); beata.dobrowolska@umlub.pl (B.D.); 5 Students’ Scientific Association, Department of Holistic Care and Nursing Management, Faculty of Health Sciences, Medical University of Lublin, 20-081 Lublin, Poland

**Keywords:** health-related behaviours, foreign students, education, diet, physical activity

## Abstract

Educational migration benefits students and receiving countries, but universities are challenged to meet a variety of needs, such as cultural adaptation and changes in health behaviours such as dietary habits. This study aimed to assess the health-related behaviours, encompassing dietary/eating habits, among international students in Poland, while also elucidating the factors influencing these behaviours. The study consisted of two phases: (phase 1) cross-sectional research among 231 foreign students using the Fantastic Lifestyle Questionnaire (FLQ); and (phase 2) focus group interviews with 15 international students. The study was conducted in accordance with STROBE (STrengthening the Reporting of OBservational studies in Epidemiology) and COREQ (COnsolidated criteria for REporting Qualitative research) Checklists. It was shown that the respondents highly value health. Students’ health-related behaviours are related to their financial situation, health condition, self-assessment of their lifestyle, the importance attached to being healthy, and their country of origin. The qualitative analysis allowed us to identify (1) the barriers related to the exhibiting of health-related behaviours, (2) expectations of foreign students regarding the exhibiting of health-related behaviours, (3) changes in the health-related behaviours, and (4) factors facilitating the exhibiting of health-related behaviours. There is a need for inter-university cooperation on a strategy to promote health-promoting behaviours of foreign students studying in Poland.

## 1. Introduction

According to the unified definition of the UNESCO (United Nations Educational, Scientific and Cultural Organization) Institute for Statistics, foreign students are individuals who have physically crossed an international border between two countries with the objective of participating in educational activities in the country of destination, where the country of destination of a given student is different from their country of origin [1]. According to data from the International Organization for Migration [2], as well as the OECD (Organisation for Economic Co-operation and Development) [3], the number of foreign students is still growing: in 2020, 4.4 million international students were studying in OECD countries, 70% more than a decade ago. The most popular destinations for foreign students are the United States, Great Britain, and Australia. China and India remain the two leading places of origin among international students [3]. Student mobility programs are most often participated in by science and technology students as well as doctoral students [3]. In Poland, the main group of foreign students comes from Ukraine, Belarus, and India [3,4]. There are also foreign students from India, Spain, Sweden, Norway, Turkey, the Czech Republic, Germany, and Taiwan [4]. Poland stands out as an attractive destination for foreigners to study due to several factors. These may include affordable tuition fees and living costs compared to other Western European countries, and a high-quality education system with numerous English-taught programs. Additionally, Poland’s central location in Europe offers easy access to other European countries, making it a convenient base for exploring the continent. For many foreign students, studying in Poland represents their first exposure to Central European cuisine and food environments. This novelty factor makes Poland an intriguing setting for studying dietary habits, as foreign students navigate and adapt to unfamiliar foods, ingredients, and meal structures, potentially influencing their dietary choices and behaviours [5].

The topic of educational migration is relevant and current, as evidenced by the number of scientific articles by various researchers from around the world on the subject [6,7,8,9,10]. The literature points to numerous driving forces for students who want to study abroad. They include a willingness to leave due to the quality of education, as well as prospects for a better career path and social status; learning a foreign language; parental influence; a desire to gain international experience; demographic influences in their own country; the accelerating process of globalization and participation in it; and the internationalization of labour markets [11,12]. Political and economic factors also motivate students to travel abroad [13]. In the case of students coming from countries affected by conflicts, educational migration is also a form of humanitarian migration [3]. Choudaha R. (2017) distinguished three waves of international student mobility caused by important events with global impacts, which shows that politics and the world economy influence the motivations for institutions to recruit foreign students, as well as of the students themselves [14]. The COVID-19 pandemic impacted student mobility, reducing it significantly at the beginning, although the available data show that the number of international students in at least half of the OECD countries has returned to pre-pandemic levels [3].

Educational migration brings a lot of benefits to students as well as the receiving countries. However, it also poses a challenge due to the acculturation process. This process is a cultural change that includes customs and psychological changes in the field of cultural identity, and these changes occur after intercultural contact [15,16]. The literature distinguishes two types of adaptation to the acculturation process. Psychological adaptation is the first of them, including good health and mental well-being. The second is socio-cultural adaptation referring to social competencies that are used in managing everyday life in an intercultural context [17]. International teaching should therefore take into account cultural sensitivity that conditions the effective acculturation process [18]. It is worth noting that health capital is a complex of health-related resources, skills, and knowledge that are key to maintaining a healthy lifestyle and coping with the challenges of academic life in a foreign country. By developing health awareness, promoting mental health, and providing access to health care services and social support, it is possible to strengthen foreign students’ health capital, which contributes to their overall well-being and academic performance [19].

The results of scientific research in recent years draw attention to the issue of changing the lifestyle of foreign students, who, after arriving in a foreign country, function in a new culture, which may have a positive or negative impact on their health-related behaviours. The risk of negative health-related behaviours occurs when these behaviours are more common in the culture of the country to which the student comes compared to their home country [6,20]. Another important risk factor is stress, which results from the acculturation process as well as from the requirements for educational programs [21,22]. Research indicates a change in the lifestyle of students in a foreign country in various areas, such as a change in eating habits in terms of eating more fast food; sweets and sweet drinks; eating meals irregularly, skipping some meals; reducing the amount of consumption of fish, vegetables, and fruits; and insufficient vitamin supplementation [23,24,25].

In the face of the widespread phenomenon of educational mobility, universities meet new challenges and the diverse needs of foreign students. The main issue is cultural adaptation and the related change in certain habits, including health-related behaviours related to diet and physical activity. There are many studies on lifestyle and health-related behaviours among foreign students representing different cultures and countries of origin [6,12,26,27,28,29]. However, the lifestyle and health-related behaviours of foreign students and the factors that influence them are mostly analysed within qualitative research. The research discussed in this work is based on a combination of quantitative and qualitative methods. Conducting research that extensively analyses this phenomenon is particularly relevant for shaping a health-promoting higher education environment for foreign students and health-promoting strategies dedicated to this group of students. Therefore, this study aimed to assess the health-related behaviours, encompassing dietary/eating habits, among international students in Poland, while also elucidating the factors influencing these behaviours using a mixed-methods methodology.

## 2. Materials and Methods

### 2.1. Aim of the Study

This study aimed to assess the health-related behaviours, encompassing dietary habits, among international students in Poland, while also elucidating the factors influencing these behaviours. Two research problems were identified:

(1) What are the health-related behaviours and dietary habits of foreign students in Poland?
(2) What are the determinants of health-related behaviours, including dietary habits, of foreign students in Poland?

### 2.2. Study Design

The research was conducted using mixed methods, comprising two phases. The first phase involved conducting cross-sectional research using a survey questionnaire, in accordance with the guidelines contained in the STROBE Checklist [30]. The second phase consisted of conducting focus group interviews with the same group of foreign students in order to obtain a better insight into the issues of health-related behaviours exhibited by foreign students in Poland. Qualitative research was reported in accordance with the COREQ Checklist [31].

### 2.3. Study Participants and Setting

Cross-sectional research was conducted in Poland among a sample of 231 foreign students of the Medical University of Lublin from 22 different countries, of which 75.3% came from Asia, 17.8% from Europe, and 6.8% from North America. Focus group interviews were conducted among a group of 15 foreign students, the vast majority of whom came from the USA.

The inclusion criteria for the study were (1) voluntary, informed consent to participate in the study, (2) studying at the Medical University of Lublin, and (3) participating in educational activities in a country different from the country of origin.

Focus group interviews were conducted with 15 foreign students. The same number of interviews were included in further analysis. The researchers decided that this number enabled data saturation to be achieved (no new information was identified during the interviews, and the content was repeated by subsequent participants) [32].

### 2.4. Research Instruments

The quantitative survey research was conducted using the Fantastic Lifestyle Questionnaire (FLQ) by Wilson et al. [33]. This tool examines the lifestyle of respondents from the last month in nine domains: F = family and friends, A = activity, N = nutrition, T = tobacco and other substances, A = alcohol, S = sleep, seat belts, and stress, T = type of personality, I = introspection, and C = career and social roles [34]. Each dimension consists of two to four items that are scored from 0 to 2 points. The total score is calculated as the sum of all items. The reliability of the scale in the original version is 0.88, and studies by other authors indicate reliability in the range of 0.68–0.92 [35,36].

Another tool was a self-designed questionnaire, which included questions about students’ sociodemographic characteristics and their financial status, health and lifestyle self-assessment, the importance of health, and changes in lifestyle at the university. The survey questions were developed on the basis of a literature review carried out by researchers. The questionnaire was pilot-tested with a small sample group to identify any ambiguities or unclear items. Feedback from this pilot test was used to refine and improve the questionnaire.

Before the qualitative research, each student received a questionnaire containing questions about sociodemographic characteristics and consent to participate in the study. During a moderated discussion, the interviewer sought to answer questions on (1) the lifestyle in the past year, (2) everyday health-related behaviours in students’ countries, (3) changes in health-related behaviours after arriving in Poland, (4) the easiest and most difficult aspects of adapting to life in a different culture or country, (5) factors that facilitate and hinder the exhibiting of health-related behaviours in a foreign country, (6) the barriers or limitations in the exhibiting of health-related behaviours in a foreign country, (7) acculturative stress, and (8) expectations towards the receiving university in supporting health-related behaviours. If necessary, supporting questions were asked to develop the discussion.

### 2.5. Data Collection Process—Quantitative Data

The survey research was conducted in 2019. Study participants were recruited using the snowball method [37]. Students were asked to identify other foreign students who might be interested in participating in the research. The paper-and-pencil method was used, and the survey questionnaires were distributed to respondents by the members of the research team. The research team included students who were members of the Scientific Club and who had previously undergone training on proper data collection. Each respondent received an explanation of the purpose of the study and the data collection procedure.

In total, 350 questionnaires were distributed to foreign students. The students returned the questionnaires in envelopes to one of the researchers’ boxes, maintaining anonymity. After checking the completeness of the questionnaires, 231 questionnaires were included for further analysis.

### 2.6. Data Collection Process—Qualitative Data

Students participating in the survey were given the opportunity to participate in in-depth focus group interviews. After explaining the purpose and procedure of the study, the students were asked to provide their email addresses to the members of the research team. An invitation for further research was sent to the provided email addresses. The interview was conducted among a group of 15 individuals who had previously given written informed consent to participate. The participants were informed about the need to record the conversation. The focus group interviews were conducted in a conference room at a round table and lasted approximately two hours. The conversation was moderated by one of the male students (M.M.) who was trained to hold the discussion. Before starting the study, each participant completed a short identification form and chose a nickname (Student 1, Student 2, etc.) to be used during the recorded conversation in order to maintain anonymity during the study. The person conducting the interviews did not have any relationship with the participants. Out of 231 respondents during the first stage of the study, 15 students expressed their willingness to participate in the second stage. During the moderated discussion, the researcher took notes used for data analysis based on a previously prepared list of open questions. The recorded interviews were transcribed using Microsoft Word 2017 without any changes to the statements and then made available to the study participants.

### 2.7. Data Analysis

The collected quantitative material was analysed with the IBM SPSS Statistics version 25 package (IBM, Kraków, Poland). Correlations between variables were examined, and the Mann–Whitney U test was performed. Data were presented as mean (M), median (Me) and standard deviation (SD). The level of significance in the analysis was *p* < 0.05.

The research material collected during the focus group interviews was subjected to thematic analysis based on the guidelines of Elo et al. (2014) in order to maintain the reliability and credibility of the results of qualitative research [38]. The thematic analysis consisted of three phases: preparation, data organization, and reporting of results [38]. Three persons from the research team independently conducted an analysis. They read the transcript several times to identify main categories and subcategories, with quotations anonymously assigned to each of them. After three independent analyses, the distinguished categories and subcategories were compared, and then the process of reconciling and unifying them was carried out jointly. In case of discrepancies, other team members were invited to the discussion to resolve doubts. The results of the analyses were presented to the research participants in order to obtain their acceptance.

### 2.8. Rigor

The rigour of the study was evaluated with the criteria Lincoln and Guba [39]. Criteria such as credibility, dependability, confirmability, transferability, and authenticity were achieved through: obtaining written informed consent, allowing participants to review the transcript of the interviews, assessment of the encoding by the research team and external check by individuals conversant in research methodology, clarification of the study method, rich description of data for a clear understanding of the study process, sampling with maximum variation, and prolonged engagement. Prolonged engagement with the participants helped the first author gain their trust and obtain a better understanding of the study fields [39].

### 2.9. Ethical Issues

Positive consent was obtained from the Bioethics Committee of the Medical University of Lublin: KE-0254/24/2018. The study was performed in accordance with the principles of the Declaration of Helsinki. Each respondent was informed about the purpose of the study and voluntary, anonymous participation as well as the possibility of withdrawing from the study at any stage. In the case of doubts, comprehensive explanations were provided. Each participant gave their written consent to participate in the study. Before the beginning of the qualitative research, the participants were informed about the need to record the conversation. Additionally, the focus group interview participants read the content of the interview transcript and then approved the analysis results in the form of selected categories.

## 3. Results

### 3.1. Study Participants—Quantitative Data

The quantitative research included 231 respondents’ answers, and more than half of the respondents were women (55.8%, *n* = 129). The mean age of the respondents was M = 21.69 (SD = 3.48). The vast majority (78.4%, *n* = 181) of the respondents declared being single, 18.2% (*n* = 42) of the individuals indicated having a partner, and the smallest group were married people (1.7%, *n* = 4). The majority of respondents assessed their financial status as good (70.6%, *n* = 163), and others as very good (21.6%, *n* = 50). The smallest group were those who indicated that the status was below average (4.8%, *n* = 11) and poor (1.3%, *n* = 3). The students most often indicated that their accommodation during the studies was dormitory (49.8%, *n* = 115) and homestay (47.6%, *n* = 110). More details are provided in Table 1.

When asked how important it was for them to be healthy in their subjective assessment, students rated health highly (Table 2). The average score on a scale from 1 to 10 was 8.78 (SD = 1.60). Assessing their health condition, students most often claimed it was good (50.2%, *n* = 116), average (30.7%, *n* = 71), and very good (11.7%, *n* = 27). When asked how they assessed their lifestyle, students most often described it as rather healthy (46.3%, *n* = 107). The majority of students claimed that student life changed their lifestyle (90.9%, *n* = 210), Table 2.

### 3.2. FLQ Scores among Foreign Students

The mean score of foreign students on the FLQ scale was M = 33.55 (SD = 6.71). The lifestyle was very good among 38.1% of foreign students (*n* = 88), good among 27.3% (*n* = 63), regular among 22.1% (*n* = 51), excellent among 9.5% (*n* = 22), and requiring improvement among 3.0% (*n* = 7) (Table 3).

The overall result of health-related behaviours measured by FLQ in the entire group of students statistically significantly correlated with the following variables: financial status (*p* = 0.000); health status (*p* = 0.000); lifestyle self-assessment (*p* = 0.000); the importance attached to being healthy (*p* = 0.001); and the country of origin (*p* < 0.001), (Table 4 and Table 5). However, individual indicators of FLQ health-related behaviours (i.e., F = family and friends, A = activity, N = nutrition, T = tobacco and other substances, A = alcohol, S = sleep, seat belts and stress, T = type of personality, I = introspection, and C = career and social roles) correlated statistically significantly with the following variables: students’ gender, financial status, health status, self-assessment of lifestyle, importance attached to being healthy, and the country of origin (Table 4 and Table 5).

### 3.3. Study Participants—Qualitative Data

Among the 15 focus group interview participants, there were 5 men and 10 women. All of them studied medicine and came from the USA, except one person from Canada. The average age of the respondents was 26 years, with the youngest student being 21 years old and the oldest 36 years old. Eight respondents were first-year students (Table 6).

### 3.4. Identified Categories

The qualitative analysis of the collected material allowed the identification of four categories related to the health determinants of the foreign students and their corresponding subcategories (Table 7): (1) barriers related to the exhibiting of health-related behaviours, (2) expectations of foreign students regarding the exhibiting of health-related behaviours, (3) changes in the health-related behaviours of foreign students after arriving in Poland, and (4) factors facilitating the exhibiting of health-related behaviours: new foreign students after arriving in Poland.

The first category refers to the barriers related to the exhibiting of health-related behaviours after arrival in Poland and includes, among others, the subcategory of the language barriers.

Foreign students reported various difficulties in everyday life related to the language barrier. They had difficulties understanding the labels on food products regarding their composition. This was especially problematic when the student had a food disease/allergy and was forced to avoid specific ingredients. Due to the lack of information in English on food products, the students were constantly afraid of consuming a product that could harm their health. In these cases, they were forced to use tools that translated each ingredient name separately into English to check the exact composition of the product.

“I have celiac disease and food allergies (…) I’m afraid I’m going to make a wrong food choice because nothing is in English, (…). I have to be very careful about what goes into my body and it’s really frustrating to have to go through every ingredient and translate it into English and figure out what it is (…)”.

(Student 1).

Students experienced limitations when using health care services due to the language difference. Since healthcare professionals were not able to communicate in English, the students were forced to search for a person providing medical services in a foreign language. The respondents also indicated that the medical staff was unhelpful and did not indicate a facility which could provide health services. The students also mentioned the medical staff at healthcare facilities were reluctant to start a conversation with them.

“Actually (…) I was with Student 1 when she had an issue with her ear and that was challenging because nobody spoke any English and they were extremely unhelpful. If she hadn’t been alright, it would have been like a disaster. We were in three different places and they were didn’t want to talk with us (…)”.

(Student 2).

Another subcategory is the organizational barriers at the university. Foreign students had difficulty locating university buildings and lecture/training rooms where classes were held; their schedule was tight and, in their opinion, poorly planned. Classes started early in the morning and lasted until late, with no breaks when they could eat, rest, or engage in physical activity. Students particularly pointed out that it was not conducive to a regular lifestyle.

“Thursday, 7 a.m. we have an anatomy lecture, then we go to have 2 h of lab followed by anatomy course and at 8.30 p.m. we have enough. I feel like that could have been revised, done differently”.

(Student 1).

The lack of a library or study spaces in some university buildings also posed a problem. The students also lacked places where they could rest between classes or eat lunch during breaks. Moreover, the students had limited access to canteens, cafes, and vending machines or shops selling water and healthy food on the campus.

“(…) in every building we should have library where we can study in between classes (…) you know you come here and there is no place to study (…). We don’t have also a place for lunch. When you are tired you can have a lunch, as well you go to the next class you know a place for lunch/library in every building will really help a lot”.

(Student 3).

The respondents pointed out the cultural barriers, including difficulties in taking up sports due to the concerns regarding social acceptance of people from a different culture. Students from African-American culture indicated that they had given up health-related behaviours, such as running, due to the lack of feeling comfortable doing this activity in the city as the only person from their culture.

“My healthy behaviour changed. I used to run on the street, just go around my neighbourhood. Here I don’t do it because I not used to see African running on the street. I haven’t tried but I just don’t feel it when I was running late (…)”.

(Student 3).

The subcategory of the climatic barriers referred to a different climate and the limitations associated with it. The students indicated that they were not used to weather conditions in which they felt constantly cold. These conditions were not conducive to undertaking activities related to taking care of health or engaging in physical activity.

“In my country I used to do yoga and hiking. I think it is not easy here because it’s cold all the time”.

(Student 4).

The barrier regarding health insurance was associated with differences in its terms and conditions and the comprehensibility of using it. Some students had not been told by the university staff how the Polish health care system worked, including the rules for the financing and the places where they could receive help.

“I’m sure if I were Polish or even European it would be simple but we used to a different system of insurance in the US and like it’s definitely like I’ve had to pay everything out of pocket and then waiting to figure out if I can get some of it back. You know, it’s not a big deal but it is confusing”.

(Student 2).

The next subcategories concerned the lack of time and limited availability of information. The lack of time resulting from the tight study schedule significantly prevented students’ physical activity and the possibility to prepare healthy, balanced meals, despite their specialised knowledge in this aspect. The students also indicated that in order to make up for vitamin deficiencies caused by an unbalanced diet, they had started taking supplements after arriving in Poland.

“(…) since I got here, I don’t cook healthy foods. I just order out all the time. I don’t have time (…), so that is Med School life and I don’t exercise often either”.

(Student 5).

“since I arrived in Poland I had to take vitamin supplements because I don’t have time to eat healthy food (…)”.

(Student 6).

In turn, the limited availability or lack of information concerned mainly the possibility of using the university sports hall, and extracurricular activities and seeking help in situations where medical advice was needed or students’ health and life were at risk.

“(…) so I don’t know about that they support us much. I also didn’t even know about counselling. I assume nobody has said about this”.

(Student 4).

“I don’t have any resources that are offered to it. I don’t know if they just don’t hand it out and let me know. Like we don’t know if they have counselling, activities for people where we can get together and work out (…) Is it available to us? We just don’t know”.

(Student 7).

The second category was the expectations of foreign students regarding the exhibiting of health-related behaviours. Subcategories were distinguished; they related to supporting students’ health-related behaviours in various aspects. Students expected support in the following areas: (1) mental health counselling in English; (2) information regarding access to health services for foreigners; (3) providing information on emergency procedures; (4) information regarding the location of health food stores; (5) organisational information and better marking of buildings and classrooms; and (6) providing a place for physical activity for students.

The students also expressed the need for a clinic with a general practitioner on the university campus where they could receive immediate advice and help in English.

“I had ear infection the other week. And I thought I might need antibiotics and finding like where to go for that? It was much more of a nightmare that I expected it to be. I do think that I wish that we had like a clinic or something on campus or at least one that I can either English and well students and like when they show up like they need you know like something that’s kind of like here for us or not running around trying to figure out what to do”.

(Student 1).

“Important I guess (…) like where to go if you have a medical emergency”.

(Student 2).

Another category was changes in the health-related behaviours of foreign students after arriving in Poland. Four subcategories were distinguished: changes in eating habits; changes in sleep patterns; experiencing stress related to acculturation; and changes in spending free time.

After arriving in the country where they studied, the students cooked less and ordered fast food more often. They justified it by lack of time due to the large number of classes at the university. They also experienced stress related to coming to a foreign country and organising their studies. The lack of knowledge and information about the culture, language, and the organisation of education in some students resulted in sleep disturbances and a reduction in its quantity and quality. The students paid attention to the changes in the way they spent their free time, ceasing to pursue their current hobbies, and giving up physical activity as a form of spending free time.

“Since, I’ve arrived to Poland, I have not been practicing my exercise as I’ve done. I have black belt, normally we do training three times a week and haven’t found a good club. So I haven’t done in my room, I was once time in the gym”.

(Student 3).

“My sleep patterns have changed a lot since coming here. I don’t know if that’s because just a result of med school but I sleep less than I used to. (…) going from America to Poland it is just like a new culture, a new language, starting med school. All of this together releases stress”.

(Student 8).

“In my country yoga and hiking. I think it is not easy here because it’s cold all the time”.

(Student 4).

“In my country I go to the gym more and go on walks regular, also on the beach”.

(Student 9).

The last separated category was factors facilitating the exhibiting of health-related behaviours of foreign students after arriving in Poland. The first sub-category was lower costs of living and easy access to products; the students were satisfied with the price of the products and the proximity of stores where they could find most food products.

“My favourite thing is our conversion rate is amazing and I love how relatively cheap everything is for us. In the US if we were had the same amount of money we’d be like scraping to get money by… because students are given like a fair loan secure like we can live very comfortably I think and here I don’t have to worry about like buying vegetables that are going to cost less dollars than US”.

(Student 1).

Another subcategory was the sense of security. The students paid particular attention to the political stability of the country, thanks to which they felt safe while staying in Poland.

“One of the other great things about Poland is it really safe at least it feels really safe (…)”.

(Student 2).

Another category referred to students’ lower stress related to higher education. The foreign students claimed that teachers were friendly towards foreigners, and, moreover, university exams did not cause as much stress as in their home country. They also paid special attention to the possibility of personal development and the possibility of taking re-sit exams.

“(…) the stress level at the medical school here is much lower compare to US. The stress in America is that if you fail three exams in one academic year you repeat the whole year. Here, it’s not the case. You can don’t pass one exam, you got another chance, you have just to become better. It is like a system, where they want you to improve and become better and just be a good person, (…) as I do exams I would do my best but if doesn’t turn out I would have another chance”.

(Student 3).

Students paid attention to factors related to healthier food. According to them, in Poland, they had access to healthier products, which were less processed or made with less salt. They also mentioned the need to walk due to the large distance between the university buildings. Thanks to the arrangement of the university infrastructure, the students had the opportunity to engage in physical activity while moving from one class to another.

“I would have to say that my healthy here is better because you’re more comfortable in the country here I am more accustomed to walking around, the food is less salty, good tasting, a lot fresher. I’ve noticed at least compared to Detroit, the food here is definitely much healthier, getting around this much healthier medical school still being active getting up every morning and going places every day but and our schedule is very busy”.

(Student 10).

“I agree with Student 10 completely. On that I think like walking every day has been like he’s really good for me and even just like in between classes like I don’t mind walking from here to maximum decorated like wakes my brain back up and it makes me feel better”.

(Student 1).

Finally, the students noticed that Poland is pet-friendly; many people had pets and one may freely walk their dog here.

“the easiest thing is that Poland is a very dog friendly (…) I can integrate into life with my doggy. Polish people love dogs”.

(Student 2).

## 4. Discussion

This study’s aim was to assess the health-related behaviours, encompassing dietary/eating habits, among international students in Poland, while also elucidating the factors influencing these behaviours. Entering into the university environment, greatly associated with starting life away from family, may result in both beneficial and risky behaviours among young people. A lot of studies on the above issues are conducted abroad. However, studies conducted with mixed methods and involving medical students are rare.

Our research showed that medical students highly valued health and described their lifestyle as rather healthy (46.3%, *n* = 107). Similar results were observed in the study by Murillo-Llorente et al., in which 51.9% of students described their lifestyle as appropriate [40]. The overall average assessment of students’ lifestyles was also obtained in Tassini et al.’s study, and, importantly, the highest score responses of “very good” and “excellent” were not recorded [41]. However, despite the general positive attitude and education in the field of healthy health-related behaviours, the students often experienced difficulties in the practical use of skills and knowledge [42]. This is confirmed by the research of Nieves et al., who showed that due to lack of time, widespread availability of convenience products and fast-food restaurants, and the lack of experience in cooking, young people developed pragmatic, fast, and nutrient-poor eating habits [43]. In Polish studies, the students expressed similar opinions, pointing out that the reason was the lack of time caused by a busy schedule. The students noticed that irregular consumption of unbalanced meals resulted from a large number of responsibilities and stress, as well as the inability to choose products that were available in their country of residence. It was also the cause of fluctuations in the students’ body weight [6,44]. It is important to note that dietary diversity can positively affect students’ academic performance. There is an observed trend that the greater the dietary diversity, the higher the educational achievement [45,46].

Additionally, the influence of peer pressure and the desire to fit in can strongly influence dietary choices. Student social gatherings often involve eating unhealthy food [47]. Moreover, the lack of appropriate education on nutrition and healthy eating habits plays a significant role in perpetuating this problem [48,49]. However, it should not be the case for the students participating in our research, as we dealt with medical students, who study medicine. A major difficulty and challenge among students, especially in the first years of study, is preparing balanced meals on their own due to poorly developed cooking skills [50]. The economic constraints that many students face also contribute to their unhealthy eating habits [51]. Financial constraints can lead to choosing cheap, processed foods over fresh and healthy alternatives. According to the surveyed students, Poland, unlike their country of origin (USA), offered relatively cheap food. As they stated in qualitative research, one could buy healthy, fresh products and vegetables cheaply. The inappropriate eating-related behaviour of foreign students can also be caused by psychological reasons: the so-called eating stress as a strategy for coping with difficult situations, e.g., the demanding academic life [52,53]. Our study also showed a relationship between a student’s country of origin and nutrition. Students usually tend to maintain eating habits from their country of origin even after migrating to a new country, however, as part of acculturation, they may develop a so-called hybrid diet [47]. Moreover, our study found no association between marital status and dietary choices, which differs from the findings of other researchers who emphasize the importance of living together with family. Existing data on the relationship between living with family and diet quality support the idea that such shared residence means better nutrition [54].

The lack of time and cultural barriers also appeared to be some of the reasons for limited physical activity among the foreign students involved in our study. Similarly, such areas as nutrition and physical activity were identified as requiring improvement in the study by Tassini et al., who noticed a great need to improve the management of students’ quality of life [41]. According to many researchers, implementing educational programs focusing on healthy eating and physical activity can be a key step in building awareness and promoting beneficial health habits among students. Additionally, investing in supporting physical activity by organizing sports activities and providing access to recreational facilities can be an effective incentive for regular exercise [55,56,57]. The students, both in our study and the studies conducted by other authors, also noticed negative changes in the quality and quantity of sleep and the level of stress [58,59]. In addition, improper quality and quantity of sleep is associated with higher caloric intake and unhealthy eating habits, so it would be worthwhile to make students aware of this aspect [60,61]. The students reported sleep disturbances and fatigue, as well as a decrease in physical activity [23,62]. The foreign students were more willing to use stimulants such as alcohol, cigarettes, or drugs [24,63]. It is due, among other things, to the fact that students are more willing to try new things in a foreign country [6], and the factors predisposing them to negative health-related behaviours are stress and experiencing negative emotions [22,64]. It is also important to create an environment conducive to mental health by providing psychological support, access to support groups, and promoting stress management techniques. Additional initiatives, such as facilitating social integration through cultural events and language courses, can help create a sense of belonging and social support among international students [65,66]. Therefore, the provision of guidance is a necessary and essential component for the effective implementation of the students into the profession [67].

Qualitative analysis of the collected material allowed us to identify four categories related to the health determinants of foreign students and their corresponding subcategories. The first category refers to the barriers related to the exhibiting of health-related behaviours after arriving in Poland and includes the following subcategories: the language barriers, the organisational barriers at the university, the cultural and climatic barriers, the barriers regarding health insurance, and the lack of time and information. Matyja A. as well as Wąsikiewicz-Firlej et al. and Kozula mention the language barrier as the most serious problem in their studies since the majority of respondents found Polish to be very difficult [68,69,70]. Moreover, Wąsikiewicz-Firlej et al. claim that some respondents, especially those from southern countries, complained about the weather [69].

In turn, the second distinguished category was the expectations of foreign students regarding the exhibiting of health-related behaviours. It should be mentioned that several respondents in Wąsikiewicz-Firlej’s study drew attention to the problems with medical care [69].

The third category was changes in the health-related behaviours of the foreign students after arriving in Poland (changes in eating habits; changes in sleep; experiencing stress; and changes in spending free time). The foreign students choose places where they can integrate with others and do not take part in organised cultural events due to their lack of knowledge of the Polish language [71]. Therefore, it was obvious that foreign students made friends and acquaintances primarily with students from other countries [69].

The last distinguished category was factors that facilitate the exhibiting of health-related behaviours by foreign students after arriving in Poland (including lower costs of living, easy access to products, sense of security, less stress related to education, healthier food, and greater activity). In his study, Popow M. proved that respondents pay more attention to the ways and methods of education at Polish universities and appreciate engaging methods and interaction with lecturers as well as a relaxed atmosphere [71]. In the study by Wąsikiewicz-Firlej et al. [69], the students particularly appreciated the high level of education and attractive educational offer, as well as the small size of class groups, which required greater activity and involvement of students. Moreover, the respondents [69] believed that Poland offered a good or even better standard of customer service than in other places. However, contrary to the results obtained in our study, Wąsikiewicz-Firlej et al. [69] claimed that several respondents assessed the local cuisine negatively. Therefore, food preferences were also different in these groups.

## 5. Conclusions

International students highly value health. It was shown that their health-related behaviours depended on their financial situation, health status, self-assessment of the lifestyle, the importance attached to being healthy, and their country of origin. Four categories related to health determinants were identified: the barriers, expectations, changes, and factors facilitating health-related behaviours. The students paid particular attention to the lack of time to prepare balanced meals, mainly due to the busy university schedule, as well as the possible negative impact of the products consumed on their health due to the inability to understand the product composition. However, they had a positive attitude towards the diversity and availability (also in terms of prices) of products, especially vegetables and fresh, unprocessed food.

Both universities receiving foreign students and public health facilities should undertake inter-university and inter-sectoral cooperation on a strategy to promote health-promoting behaviours among foreign students studying in Poland. Educational migration is a growing trend, not only in Poland but also across the world; therefore, such strategies are important on a global scale, not only locally. 

## 6. Limitations and Directions for Future Research

Several limitations can be identified in this study. The first was the uneven diversity of the students in terms of their country of origin: in the quantitative research, students represented 22 countries, the largest part of which were students from Asia. Another limitation was the selection of the study group, which was not random. The study group consisted of students from only one university and one field of study. Additionally, the qualitative research involved students only from the USA (with one exception), which also limits the generalisability of the conclusions. Moreover, the FLQ may lack cultural sensitivity or appropriateness for all populations, particularly if language or cultural differences impact questions understanding or interpretation as our study group include foreign students from different countries for whom the English language has not always been their native language. In addition, we recommend future research comparing health behaviours and factors determining them among foreign students studying in several countries, e.g., European ones, selected based on the popularity of these countries among migrating students. Such research, using a mixed methodology, would establish some universal trends for health promotion strategies among migrant students at the macro level.

## Figures and Tables

**Table 1:** Descriptive Statistics.

Variables		M	SD
Age		21.69	3.48
		*n*	%
Sex	Female	129	55.8
	Male	101	43.7
	ND	1	0.4
Marital status	Single	181	78.4
	Cohabitating	42	18.2
	Married	4	1.7
	ND	4	1.7
Financial status	Very good	50	21.6
	Good	163	70.6
	Below average	11	4.8
	Rather poor	3	1.3
	ND	4	1.7
Place of residence	Dormitory	115	49.8
	Homestay	110	47.6
	Other	4	1.7
	ND	2	0.9

M—mean, SD—standard deviation, ND—no data.

**Table 2:** Student lifestyles.

Variables		Mean [Scale 1–10]	SD
Importance of being healthy		8.78	1.60
		*n*	%
Health condition	Very good	27	11.7
	Good	116	50.2
	Fair	71	30.7
	Bad	11	4.8
	Very bad	3	1.3
	ND	3	1.3
Style of life	Definitely healthy	24	10.4
	Rather healthy	107	46.3
	Difficult to say	74	32.0
	Rather unhealthy	17	7.4
	Definitely unhealthy	6	2.6
	ND	3	1.3
Student life has changed your lifestyle	Yes	210	90.9
	No	18	7.8
	ND	3	1.3

SD—standard deviation, ND—no data.

**Table 3:** FLQ scores among international students.

FLQ	*n*	%
Excellent	22	9.5
Very good	88	38.1
Good	63	27.3
Regular	51	22.1
Requiring improvement	7	3.0

**Table 4:** Determinants of FLQ health-related behaviours.

Variable	Statistics										
	FLQ	F	A	N	T	A	S	T	I	C	Global
**Gender**	Z	**−2.937**	**−2.357**	−1.271	−1.362	**−1.989**	−1.100	−1.143	**−2.357**	**−2.360**	−0.096
	*p*	**0.003**	**0.018**	0.204	0.173	**0.047**	0.271	0.253	**0.018**	**0.018**	0.924
**Material situtation**	H	**14.980**	4.820	3.520	3.452	1.284	**12.500**	**7.753**	**15.140**	**17.655**	**17.335**
	*p*	**0.001**	0.090	0.172	0.178	0.526	**0.002**	**0.021**	**0.001**	**0.000**	**0.000**
**Health condition**	H	**6.852**	**26.472**	**15.815**	0.627	1.038	**17.597**	2.857	**15.347**	**12.804**	**25.499**
	*p*	**0.033**	**0.000**	**0.000**	0.731	0.595	**0.000**	0.240	**0.000**	**0.002**	**0.000**
**Style of life**	H	5.174	**27.622**	**25.141**	3.515	0.102	**17.897**	**4.271**	**9.117**	**11.307**	**31.588**
	*p*	0.075	**0.000**	**0.000**	0.172	0.950	**0.000**	**0.118**	**0.010**	**0.004**	**0.000**
**Importance of being healthy**	rho	**0.136**	**0.164**	**0.134**	0.006	0.086	0.069	0.057	**0.220**	**0.147**	**0.221**
	*p*	**0.040**	**0.014**	**0.044**	0.923	0.196	0.298	0.395	**0.001**	**0.027**	**0.001**

Z—Mann-Whitney U test, H—Kruskal–Wallis test, rho—Spearman’s rank correlation coefficient, bold—statistically significantly correlation.

**Table 5:** Country of origin vs. FLQ.

Groups		Global	F	A	N	T	A	S	T	I	C
Central Europe	M	36.26	5.11	2.15	5.45	4.84	3.84	3.82	3.74	4.16	3.15
	SD	6.21	1.23	1.09	1.71	1.44	0.51	1.15	1.29	1.44	0.90
Western Europe	M	38.29	4.71	2.43	5.43	5.57	3.71	4.14	4.43	4.57	3.29
	SD	5.02	1.11	0.79	1.51	0.79	0.49	0.90	1.40	1.40	0.76
Northern Europe	M	32.31	5.22	1.88	4.00	4.69	3.81	2.38	3.75	3.63	2.97
	SD	7.31	1.21	0.98	2.18	1.45	0.64	1.60	1.67	1.70	1.00
Northern America	M	29.60	3.93	2.53	3.80	4.73	3.00	2.67	3.20	3.20	2.53
	SD	8.10	1.33	1.06	1.74	1.49	1.25	2.06	1.78	1.78	1.25
Eastern and Southern Asia	M	35.17	4.77	2.33	4.73	5.34	3.74	3.71	3.74	3.85	2.95
	SD	5.00	1.33	1.13	1.59	0.97	0.56	1.33	1.22	1.36	0.94
Central and Western Asia	M	33.02	4.40	2.41	4.80	5.06	3.43	3.24	3.59	3.54	2.55
	SD	7.20	1.45	1.12	1.92	1.39	0.91	1.47	1.31	1.31	0.95
Middle East/Africa	M	32.13	4.25	2.25	4.75	4.63	3.88	3.00	3.13	3.63	2.63
	SD	8.72	1.67	1.04	1.91	1.69	0.35	1.93	1.55	1.19	1.51
Statistics	H	**33.817**	**30.713**	8.504	**32.198**	11.022	**37.767**	**36.407**	5.587	**20.389**	**27.734**
	*p*	**<0.001**	**<0.001**	0.203	**<0.001**	0.088	**<0.001**	**<0.001**	0.471	**0.002**	**<0.001**

M—mean, SD—standard deviation, H—Kruskal–Wallis test, bold—statistically significantly correlation.

**Table 6:** Characteristics of focus group participants.

Student Code	Age	Sex	Field of Study	Year of Study	Nationality	Country of Residence	Studying Abroad: First Time/Another Time
1	29	F	Medicine	3	American	USA	First
2	36	F	Medicine	3	American	USA	First
3	31	M	Medicine	3	American	USA	First
4	24	F	Medicine	1	American	USA	First
5	26	F	Medicine	1	American	USA	First
6	25	F	Medicine	3	American	USA	First
7	32	F	Medicine	3	American	USA	First
8	23	F	Medicine	1	American	USA	First
9	28	M	Medicine	3	American	USA	First
10	27	M	Medicine	1	American	USA	First
11	21	F	Medicine	1	American	USA	First
12	22	F	Medicine	1	American	USA	First
13	24	M	Medicine	1	American	USA	First
14	22	F	Medicine	3	Canadian	Canada	First
15	24	M	Medicine	1	American	USA	First

**Table 7:** Categories and subcategories identified as a result of the analysis of the research material.

Categories	Subcategories
Barriers related to the exhibiting of health-promoting behaviours after arrival.	Language barriers
	Organisational barriers at the university
	Cultural barriers
	Climate barriers
	Health insurance barrier
	Lack of time
	Lack of information
Expectations of foreign students regarding the exhibiting of health-related behaviours.	Health care system
	Information
Changes in the health-related behaviours of foreign students after arriving in Poland.	Changes in eating habits
	Changes in physical activity
	Changes in the quantity and quality of sleep
	Acculturation stress
	Changes in spending free time
Factors facilitating the exhibiting of health-related behaviours among foreign students after arriving in Poland.	Lower costs of living, easy access to products
	Sense of security
	Less stress related to studying
	Healthier food and walking a lot
	A pet-friendly country

## Data Availability

All data generated and analysed during the current study are not publicly available in order to protect participants’ privacy and confidentiality, but are available from the corresponding author on reasonable request.

## References

[1] UNESCO Institute for Statistics. https://glossary.uis.unesco.org/glossary/en/home.

[2] GLOBAL MIGRATION INDICATORS International Organization for Migration Global Migration Data Analysis Centre (GMDAC) International Organization for Migration, Berlin, Germany 2018. https://publications.iom.int/system/files/pdf/global_migration_indicators_2018.pdf.

[3] International Migration Outlook 2022 International Students: A Growing Group of Migrants in the OECD. https://read.oecd-ilibrary.org/social-issues-migration-health/international-migration-outlook-2022_ec0742a4-en#page4.

[4] “Foreign Students in Poland 2019” Report Study in Poland. https://www.studyinpoland.pl/en/news/82-foreign-students-in-poland-2019.

[5] Lipowski M. Experience of Erasmus students studying in Poland. Knowledge and Learning: Global Empowerment. Proceedings of the Management, Knowledge and Learning International Conference International School for Social and Business Studies.

[6] Yan Z., Fitz Patrick K. (2016). Acculturation and health behaviors among international students: A qualitative approach. Nurs. Health Sci..

[7] Alakaam A., Willyard A. (2020). Eating habits and dietary acculturation effects among international college students in the United States. AIMS Public Health.

[8] Martins J.M.S., Ferreira E.A.L., Valete C.O.S., Gramasco H.H.F. (2022). Fantastic Lifestyle Questionnaire applied to undergraduate medical students during the COVID-19 pandemic: A factor analysis. Rev. Assoc. Med. Bras..

[9] Andraus G.S., Vieira F.M., Candido G.M., Patino G.P., Bernardelli R.S., de Palma H.L.A. (2023). Associations between Lifestyle and Sociodemographic Factors in Medical Students: A Cross Sectional Study. J. Lifestyle Med..

[10] Ramírez-Vélez R., Triana-Reina H.R., Carrillo H.A., Ramos-Sepúlveda J.A., Rubio F., Poches-Franco L., Rincón-Párraga D., Meneses-Echávez J.F., Correa-Bautista J.E. (2015). A cross-sectional study of Colombian University students’ self-perceived lifestyle. SpringerPlus.

[11] Griner J., Sobol A. (2014). Chinese Students’ Motivations for Studying Abroad. Glob. Stud. J..

[12] King R., Sondhi G. (2018). International student migration: A comparison of UK and Indian students’ motivations for studying abroad. Glob. Soc. Educ..

[13] Teichler U. (2017). Internationalisation Trends in Higher Education and the Changing Role of International Student Mobility. J. Int. Mobil..

[14] Choudaha R. (2017). Three waves of international student mobility (1999–2020). Stud. High. Educ..

[15] Berry J.W., Chun K., Balls-Organista P., Martin G. (2003). Conceptual approaches to acculturation. Acculturation: Advances in Theory, Measurement, and Applied Research.

[16] Phinney J.S., Chun K.M., Organista P.B., Marín G. (2003). Ethnic identity and acculturation. Acculturation: Advances in Theory, Measurement, and Applied Research.

[17] Berry J.W., Phinney J.S., Sam D.L., Vedder P. (2006). Immigrant youth: Acculturation, identity, and adaptation. Appl. Psychol..

[18] Bannier B.J. (2016). Global trends in transnational education. Int. J. Inf. Educ. Technol..

[19] Schneider-Kamp A. (2021). Health capital: Toward a conceptual framework for understanding the construction of individual health. Social. Theory Health.

[20] Berry J.W. (2005). Acculturation: Living successfully in two cultures. Int. J. Intercult. Relat..

[21] Socolov S., Iorga M., Munteanu C., Ioan B.G. (2017). Acculturation and stress among international students. Stud. UBB Bioethica.

[22] Malau-Aduli B.S. (2011). Exploring the experiences and coping strategies of international medical students. BMC Med. Educ..

[23] Haq I., Mariyam Z., Li M., Huang X., Jiang P., Zeb F., Wu X., Feng Q., Zhou M. (2018). A Comparative Study of Nutritional Status, Knowledge Attitude and Practices (KAP) and Dietary Intake between International and Chinese Students in Nanjing, China. Int. J. Environ. Res. Public Health.

[24] Loomes S., Croft A. (2013). An investigation into the eating behaviour of international students studying at an Australian university: Should we be concerned?. J. High. Educ. Pol. Manag..

[25] Cebirbay M., Aktas N., Calderoni M. (2011). Determination of breakfast habits and knowledge of foreign undergraduates studying at Selcuk University in Turkey. Prog. Nutr..

[26] Machul M., Bieniak M., Chałdaś-Majdańska J., Bąk J., Chrzan-Rodak A., Mazurek P., Pawłowski P., Makuch-Kuśmierz D., Obuchowska A., Bartoszek A. (2020). Lifestyle Practices, Satisfaction with Life and the Level of Perceived Stress of Polish and Foreign Medical Students Studying in Poland. Int. J. Environ. Res. Public Health.

[27] Yang X.Y., Yang F.J. (2018). Acculturation versus Cultural Retention: The Interactive Impact of Acculturation and Co-ethnic Ties on Substance Use Among Chinese Students in the United States. Immigr. Minor. Health.

[28] Skromanis S., Cooling N., Rodgers B., Purton T., Fan F., Bridgman H., Harris K., Presser J., Mond J. (2018). Health and Well-Being of International University Students, and Comparison with Domestic Students, in Tasmania, Australia. Int. J. Environ. Res. Public Health.

[29] Zalewska-Puchała J., Majda A., Bożek J. (2014). Health behaviours of the students from Norway studying in Poland. Nurs. Probl..

[30] Von Elm E., Altman D.G., Egger M., Pocock S.J., Gøtzsche P.C., Vandenbroucke J.P., STROBE Initiative (2014). The Strengthening the Reporting of Observational Studies in Epidemiology (STROBE) Statement: Guidelines for reporting observational studies. Int. J. Surg..

[31] Tong A., Sainsbury P., Craig J. (2007). Consolidated criteria for reporting qualitative research (COREQ): A 32-item checklist for interviews and focus groups. Int. J. Qual. Health Care.

[32] Saunders B., Sim J., Kingstone T., Baker S., Waterfield J., Bartlam B., Burroughs H., Jinks C. (2018). Saturation in qualitative research: Exploring its conceptualization and operationalization. Qual. Quant..

[33] Wilson D.M., Ciliska D. (1984). Lifestyle Assessment. Can. Fam. Physician.

[34] Wilson D., Nielsen E., Ciliska D. (1984). Lifestyle Assessment: Testing the FANTASTIC Instrument. Can. Fam. Phys..

[35] Wilhelm K., Handley T., Reddy P. (2016). Exploring the validity of the Fantastic Lifestyle Checklist in an inner city population of people presenting suicidal behaviours. Aust. N. Z. J. Psychiatry.

[36] Deluga A., Kosicka B., Dobrowolska B., Chrzan-Rodak A., Jurek K., Wrońska I., Ksykiewicz-Dorota A., Jędrych M., Drop B. (2018). Lifestyle of the elderly living in rural and urban areas measured by the FANTASTIC Life Inventory. Ann. Agric. Environ. Med..

[37] Etikan I., Alkassim R., Abubakar S. (2016). Comparison of snowball sampling and sequential sampling technique. Biom. Biostat. Int. J..

[38] Elo S., Kääriäinen M., Kanste O., Pölkki T., Utriainen K., Kyngäs H. (2014). Qualitative Content Analysis. SAGE Open.

[39] Lincoln Y.S., Guba E.G. (1985). Naturalistic Inquiry.

[40] Murillo-Llorente M.T., Brito-Gallego R., Alcalá-Dávalos M.L., Legidos-García M.E., Pérez-Murillo J., Perez-Bermejo M. (2022). The Validity and Reliability of the FANTASTIC Questionnaire for Nutritional and Lifestyle Studies in University Students. Nutrients.

[41] Tassini C.C., Val GRd Candido S.d.S., Bachur C.K. (2017). Assessment of the Lifestyle of University Students in the Healthcare Area Using the Fantastic Questionnaire. Int. J. Cardiovasc. Sci..

[42] Hsieh H.F., Shannon S.E. (2005). Three approaches to qualitative content analysis. Qual. Health Res..

[43] López Nieves G., Sosa Cordobés E., Garrido Fernández A., Travé González G., García-Padilla F.M. (2019). Habits, preferences and culinary skills of first-year students at the university of Huelva. Enferm. Glob..

[44] Choi Y.J., Hwang J., Yi J. (2011). Acculturation, Body Perception, and Weight Status among Vietnamese American Students. J. Immigr. Minor. Health.

[45] Burrows T., Goldman S., Pursey K., Lim R. (2017). Is there an association between dietary intake and academic achievement: A systematic review. J. Hum. Nutr. Diet..

[46] Bouchefra S., El Ghouddany S., Ouali K., Bour A. (2023). Is good dietary diversity a predictor of academic success?. Acta Biomed..

[47] Shi Y., Lukomskyj N., Allman-Farinelli M. (2021). Food access, dietary acculturation, and food insecurity among international tertiary education students: A scoping review. Nutrition.

[48] Poobalan A.S., Aucott L.S., Clarke A., Smith W.C. (2014). Diet behaviour among young people in transition to adulthood (18–25-year-olds): A mixed method study. Health Psychol. Behav. Med..

[49] Kabir A., Miah S., Islam A. (2018). Factors influencing eating behavior and dietary intake among resident students in a public university in Bangladesh: A qualitative study. PLoS ONE.

[50] Hilger J., Loerbroks A., Diehl K. (2017). Eating behaviour of university students in Germany: Dietary intake, barriers to healthy eating and changes in eating behaviour since the time of matriculation. Appetite.

[51] Zarei M., Taib M.N., Zarei F. (2013). Lifestyle factors and dietary intake of Iranian postgraduate students at Universiti Putra Malaysia (UPM). Electron. Physician.

[52] Michels N., Man T., Vinck B., Verbeyst L. (2020). Dietary changes and its psychosocial moderators during the university examination period. Eur. J. Nutr..

[53] Yönder Ertem M., Karakaş M. (2021). Relationship between emotional eating and coping with stress of nursing students. Perspect. Psychiatr. Care.

[54] Martinez-Lacoba R., Pardo-Garcia I., Amo-Saus E., Escribano-Sotos F. (2018). Socioeconomic, demographic and lifestyle-related factors associated with unhealthy diet: A cross-sectional study of university students. BMC Public Health.

[55] Pop L.-M., Iorga M., Muraru I.-D., Petrariu F.-D. (2021). Assessment of Dietary Habits, Physical Activity and Lifestyle in Medical University Students. Sustainability.

[56] Plotnikoff R.C., Costigan S.A., Williams R.L., Hutchesson M.J., Kennedy S.G., Robards S.L., Allen J., Collins C.E., Callister R., Germov J. (2015). Effectiveness of interventions targeting physical activity, nutrition and healthy weight for university and college students: A systematic review and meta-analysis. Int. J. Behav. Nutr. Phys. Act..

[57] Javed A., Ameer S., Talib H., Bashir I., Jamshaid M. (2021). Evaluating Concept of Healthy Eating in Relation with Physical Activity And Dietary Habits Among University Students: Healthy Eating in Relation with Physical Activity and Dietary Habits. Pak. Armed Forces Med. J..

[58] Loureiro E., McIntyre T., Mota-Cardoso R., Ferreira M.A. (2008). The relationship between stress and life-style of students at the Faculty of Medicine of Oporto. Acta Med. Port..

[59] López-Carmona J., Rodríguez-Moctezuma R., Munguía-Miranda C., Hernández-Santiago J., de la Torre E.C. (2000). Validity and reliability of FANTASTIC an instrument for measuring the lifestyle in Mexican patients with arterial hypertension. Aten. Prim..

[60] Frank S., Gonzalez K., Lee-Ang L., Young M.C., Tamez M., Mattei J. (2017). Diet and Sleep Physiology: Public Health and Clinical Implications. Front. Neurol..

[61] Doo M., Wang C. (2020). Associations among Sleep Quality, Changes in Eating Habits, and Overweight or Obesity after Studying Abroad among International Students in South Korea. Nutrients.

[62] Eliasson A.H., Eliasson A.H., Lettieri C.J. (2017). Differences in sleep habits, study time, and academic performance between US-born and foreign-born college students. Sleep. Breath..

[63] Aresi G., Moore S., Marta E. (2016). Italian credit mobility students significantly increase their alcohol intake, risky drinking and related consequences during the study abroad experience. Alcohol Alcohol..

[64] Lu H. (2015). Burgers or Tofu? Eating Between Two Worlds: Risk Information Seeking and Processing during Dietary Acculturation. Health Commun..

[65] Kristiana I.F., Karyanta N.A., Simanjuntak E., Prihatsanti U., Ingarianti T.M., Shohib M. (2022). Social Support and Acculturative Stress of International Students. Int. J. Environ. Res. Public Health.

[66] Razgulin J., Argustaitė-Zailskienė G., Šmigelskas K. (2023). The role of social support and sociocultural adjustment for international students’ mental health. Sci. Rep..

[67] Gotlib J., Cieślak I., Jaworski M., Witkowska-Zimny M., Małkowski PPanczyk M. (2022). “Truly once in a lifetime opportunity”. An overview of nursing students experiences of study-to-work transition in the SARS-CoV-2 pandemic. Nurs. 21st Century.

[68] Matyja A. (2018). Studenci polscy i zagraniczni–specyfika ich relacji na Przykładzie Uniwersytetu Warszawskiego. Eduk. Międzynarodowa.

[69] Wąsikiewicz-Firlej E., Szczepaniak-Kozak A., Lankiewicz H. (2022). Doświadczenie Pobytu w Polsce w Narracjach Zagranicznych Studentów.

[70] Kozula M. (2018). Adaptacja studentów zagranicznych na uczelniach jako główne wyzwanie edukacji migracyjnej w Polsce. Kult. Społeczeństwo Eduk..

[71] Popow M. (2015). Polska jako miejsce studiowania w doświadczeniach studentów zagranicznych. Forum Oświatowe.

